# Perceptions of pregnant women on antenatal care visit during their first trimester at area 25 health center in Lilongwe, Malawi – a qualitative study

**DOI:** 10.1186/s12905-023-02800-7

**Published:** 2023-12-04

**Authors:** Modesta Nyando, Dziwenji Makombe, Alexander Mboma, Elias Mwakilama, Lot Nyirenda

**Affiliations:** 1 School of Global and Public Health, Kamuzu University of Health Sciences, Blantyre, Malawi; 2 Malawi Ministry of Health and Population Services, Lilongwe District Health Office, Lilongwe, Malawi; 3 Department of Community Health Nursing, Kamuzu University of Health Sciences, Lilongwe, Malawi; 4 Department of Midwifery, Kamuzu University of Health Sciences, Lilongwe, Malawi; 5 Department of Mathematical Sciences, University of Malawi, Zomba, Malawi

**Keywords:** First trimester, Pregnant women, Perceptions, Antenatal care, Malawi

## Abstract

**Background:**

Initiation of antenatal care during the first trimester is crucial for reducing maternal and neonatal morbidity and mortality. Unfortunately, only 24% of pregnant women in Malawi initiate antenatal care during this time with even lower rates of 15% at Area 25 Health Centre in Lilongwe. Despite such cases, there is little literature on obstacles that prevent women from accessing first-trimester antenatal care in Malawi.

**Aim:**

To explore perceptions of pregnant women and how they influence antenatal care visits during the first trimester at Area 25 Health Centre in Lilongwe, Malawi.

**Methods:**

We employed a qualitative exploratory study on 55 purposely identified participants. The participants were aged between 18 and 37 years with a gestational period of 36 weeks and below and attended antenatal care at Area 25 Health Centre in Lilongwe Urban, Malawi. Data were collected by MN and 2 data collectors from 19th March 2021 to 16th April 2021 through a total of 15 In-depth Interviews (IDIs) and four Focus Group Discussions (FGDs). Data were manually analysed using thematic analysis, which included categorization and deductive theme identification with reference to the study objectives and the Health Belief Model (HBM).

**Results:**

Pregnant women perceived that the first-trimester antenatal care visits were only for those experiencing ill health conditions like backache, headache, and HIV/AIDS during pregnancy. First-trimester pregnancy was perceived as too small and not worthy of seeking antenatal care; the women placed a low value on it. The majority of those who initiated antenatal care in the first trimester had previously experienced disorders and complications such as previous cesarean sections and abortions. In addition to limited knowledge about the required total number of ANC visits, challenges such as long-distance, preoccupation with business, multiple antenatal visits, scheduling of antenatal care visits, negative attitude of health workers, adherence to COVID-19 containment measures, and inadequate partner support, were identified as barriers to seeking antenatal care during the first trimester.

**Conclusion:**

The negative perceptions among pregnant women, coupled with various health systems, socio-economic and individual barriers, contributed to low attendance rates for first trimester antenatal care in Malawi. Addressing knowledge gaps and overcoming barriers related to economic, individual and health care delivery can improve women’s early antenatal care visits. Future research should consider the pregnant women from diverse socioeconomic backgrounds to gain a better understanding of these perceptions and barriers.

## Background

Starting antenatal care (ANC) in first trimester (0 to 12 weeks gestation) is one of the key interventions to reduce maternal and neonatal morbidity and mortality ratios, as it helps early identification of high risk pregnancies and educates women for positive pregnancy and labor outcomes [1]. Globally, the Maternal Mortality Ratio (MMR) is high at 303000/ 100000 live births while 546 deaths per 100,000 live births occur in sub-Saharan Africa (SSA) [2]. Malawi is among the SSA countries with high MMR reported at 439 deaths/100,000 live births and a Neonatal Mortality Ratio (NMR) of 27/1000 live births [3].

Evidence indicates that the initiation of antenatal care in the first trimester, coupled with eight contact visits, improves positive pregnancy outcomes [4].The World Health Organization (WHO) recommends that pregnant women should initiate antenatal care in the first trimester with a total of eight contact visits [5]. Despite the significant benefits of early detection and management of pregnancy related complications during ANC visits [6], not all pregnant women in Malawi initiate antenatal care in the first trimester.

According to the Malawi Demographic Health Survey (MDHS) [3], only 24% of pregnant women start ANC in the first trimester [3]. Furthermore, data from the Lilongwe District Health Management Information system [7] indicate that in the year 2018, only 15% of pregnant women who attended ANC services at Area 25 health centre did so in their first trimester [7]. Late initiation of ANC poses risks as they may delay diagnosis of pregnancy related complications, such as anemia and pre-eclampsia, which can negatively affect maternal and fetal wellbeing [3, 8, 9]. According to the United Nations (UN) Sustainable Development Goal (SDG) number 3, MMR and NMR have to be reduced to less than 70 per 100,000 live births and 12 per 1000 live births by 2030, respectively [10].

Pregnant women’s perceptions towards attending ANC in the first-trimester significantly influence the utilization of antenatal services and hence pregnancy outcomes [11]. However, there is lack of understanding on how these perceptions influence attendance of the ANC during the first-trimester. To our knowledge, there is limited literature on qualitative studies that have tried to understand the perceptions of pregnant women towards attending ANC in their first-trimester in Malawi. Therefore, this study aimed at exploring the pregnant women’s perceptions towards antenatal care attendance during their first-trimester and examine the connection between these perceptions and actual attendance. This study employs The Health Belief Model (HBM) as a theoretical framework and makes a valuable contribution to this study by identifying appropriate strategies to enhance first-trimester attendance in Malawi, ultimately reducing preventable maternal and neonatal deaths in SSA.

### The health belief model (HBM)

**Fig. 1:**
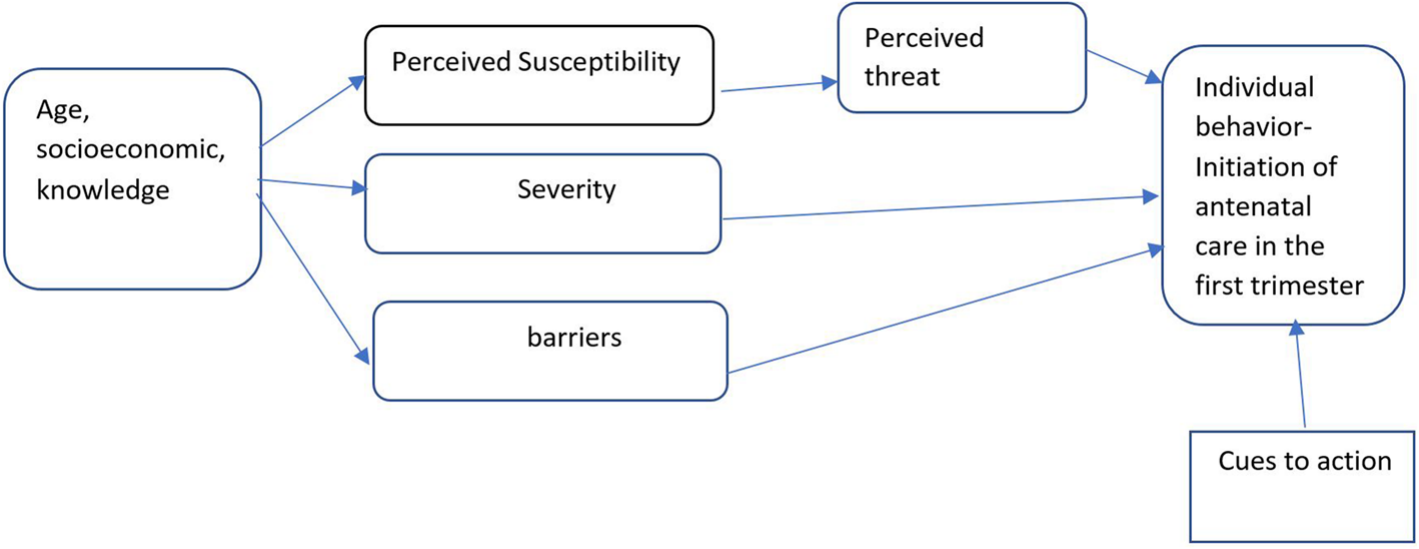
Theoretical framework of the Health belief model as adapted and applied to perceptions of women towards antenatal care during the first semester in Malawi [12]

We employed the HBM by Becker (1974) to guide the framework of exploring pregnant women’s perceptions towards attending ANC in the first-trimester. Perceived susceptibility, perceived severity, perceived threat and perceived barriers were the constructs which we used [12] (Fig. 1).

### Application of health belief model

1. Perceived susceptibility: We used the HBM to understand whether pregnant women thought their failure to attend ANC during first trimester (0–12 weeks of gestation) could affect them. Thus, whether women felt susceptible to dangers that come with failure to attend the first trimester such as, late diagnosis of complications, which include anemia and premature births [13]. We anticipated that the greater the women perceived such risk, the greater the women’s likelihood of engaging in the aimed behaviors to decrease the risk [12].
2. Severity: When pregnant women recognize that they are susceptible to getting an ill condition, it does not motivate them to take necessary action until they appreciate that getting the condition has serious physical, psychological, and social implications for themselves and their babies. In this study, we anticipated that pregnant women with previous and current complications such as previous caesarian section, and backache, respectively, would view themselves to be at greatest risk of severe complications, which would trigger them to start ANC in the first trimester.
3. Perceived threat: Pregnant women were assessed on how they rated the threat that comes with not attending ANC in the first trimester. According to the HBM, women would not participate in preventative health care programs unless they viewed themselves as potentially vulnerable and the threat of the problem as serious.
4. Perceived barriers: Perceived barriers can affect women’s decision-making. Here, we searched for the barriers that women faced or anticipated to face in relation to attending antenatal care in the first trimester.

## Methods

### Design

This study utilized exploratory qualitative research methods. We chose this design because of its ability to unfold and have an in-depth understanding of pregnant women’s perceptions and experiences towards first trimester antenatal care attendance [14, 15]. The study achieved this through in-depth interviews (IDIs) and focus group discussions (FGDs).

### Study setting and population

We conducted the study at Area 25 antenatal clinic in Lilongwe urban district in Malawi. It is an urban government Primary Health Centre, which is located 20 kms North of Lilongwe district in Malawi. All the services at the center are provided for free. At the time of the study, the Health Centre had a total catchment population of 222,054 with 11, 102 expected pregnancies [16]. The center attends to new visits on Tuesdays and Thursdays while Mondays, Wednesdays and Fridays are for subsequent visits. It sees an average of 700 new visits every month [7]. The facility provides other services such as labour and delivery, postnatal care, family planning, and outpatient services. The site was chosen because of the high volume of pregnant women with low attendance in the first trimester at 15% [7], compared with the national rate at 24% [3]. The site was also one of the facilities providing the eight antenatal contact model recommended by WHO in 2016. In addition, the selection of the facility also considered feasibility, convenience and cost effectiveness of implementing this study considering that the first author worked at the facility at the time of the study. The facility has 107 health care workers, of which 10 are assigned to the antenatal clinic.

### Sampling and sample size

The study used a purposeful sampling technique to identify study participants. We employed a maximum variation of purposively sampled population to inform the research questions^14^. This was to obtain valuable insights on the respondents’ perceptions, experiences and attitudes in relation to the aim of the study. To achieve this, an inclusion-exclusion criteria was thus employed.

First, the inclusion criteria was used: a) pregnant women aged 18 years and above; b) confirmed pregnancy through physical examination, pregnancy test or ultra sound scan; c) gestation of 36 weeks and below; d) pregnant women attending antenatal care at the clinic; e) pregnant women who had consented for the study; f) pregnant women in the first trimester and above; g) pregnant women who came for the initial and subsequent antenatal care visits, and h) primigravidas and multigravidas from all the three trimesters of pregnancy regardless of their educational,marital status and occupation. Primigravidas were women with their first pregnancy, while multigravidas were women who had been pregnant more than once. Then, an exclusion criteria was used: a) sick pregnant women, b) pregnant women above 36 weeks as they could not withstand interviews because of their condition, c) pregnant women less than 18 years, and d) pregnant women who were not willing to participate in the study. Those who refused to participate were allowed to proceed with routine activities and were reassured that their refusal would not affect their accessibility and utilization of services at the health facility. Hence, a total of 55 respondents were identified at the time of the study.

### Data collection

The first author (MN) and two assistants (midwives**)** collected data from 19th March 2021 to 16th April, 2021. MN, who had supervisory and data collection roles oriented the assistants to the study protocol and data collection process. The first author and one assistant were female while another assistant was male. The team collected data face-to-face through IDIs and FGDs using the interview guide, which was pretested on three pregnant women at Area 18 Urban Health Centre, Lilongwe district in Malawi. Pretesting of data collection tools assisted to check if the interview guide was to provide the required information of the study [17] and strengthened probing skills.

During the data collection exercise, two approaches were used on the 55 identified respondents; Indepth Interviews (IDIs) and Focus Group Discussions (FGDs). During IDIs, the data collection team solicited participants as the latter left the medical examination rooms. The team conducted the interviews, which they audio-recorded, in *Chichewa*, a local language in Malawi. They collected the data until saturation point, which was arrived at IDI no 13. Nonetheless, two more IDIs were added to 13 IDIs to take care of the sub-sampling error even if the additional two interviews did not result in identification of new ideas. Thus a total of 15 IDIs were conducted.

To conduct the FGDs, we had two separate groups; for primigravidas and gravida 2 on one hand, and Gravida 3 and above on the other hand. We also had separate groups for pregnant women who came for their initial visits and pregnant women who came for the subsequent visits. These separation processes were done to ensure that women express their views freely, and that the perceived pregnancy experiences should not be dominated by a single category of women. At the time of data collection using the FGD approach, the team reached data saturation in the midst of FGD number four and stopped data collection at the end of that FGD. Thus, the reminaing 40 respondents participated in the 4 FGDs.

Despite collecting data in a context of the COVID-19 the team conducted the IDIs and FGDs in a face-to-face, in-person, format while taking all the precautionary measures. On average, both interviews and discussions lasted for about 45 to 60 min.

### Data analysis

We analyzed data manually, guided by the six phases of thematic analysis as proposed by Braun and Clark [18]. The analysis was done by MN, DM, AM, EM and LN. These phases were (1); familiarizing self with data (2), generating initial codes (3), searching for themes (4), reviewing potential themes (5), defining and naming themes, and (5) producing a report. To complete this analytical process, a theory driven code book was developed by MN and LN, which was used to categorize and reduce raw data. Overall, the HBM constructs (Fig. 1) guided the analysis, and development of some of the initial codes, including perceived dangers and barriers, which emanated from such constructs.

**Fig. 2:**
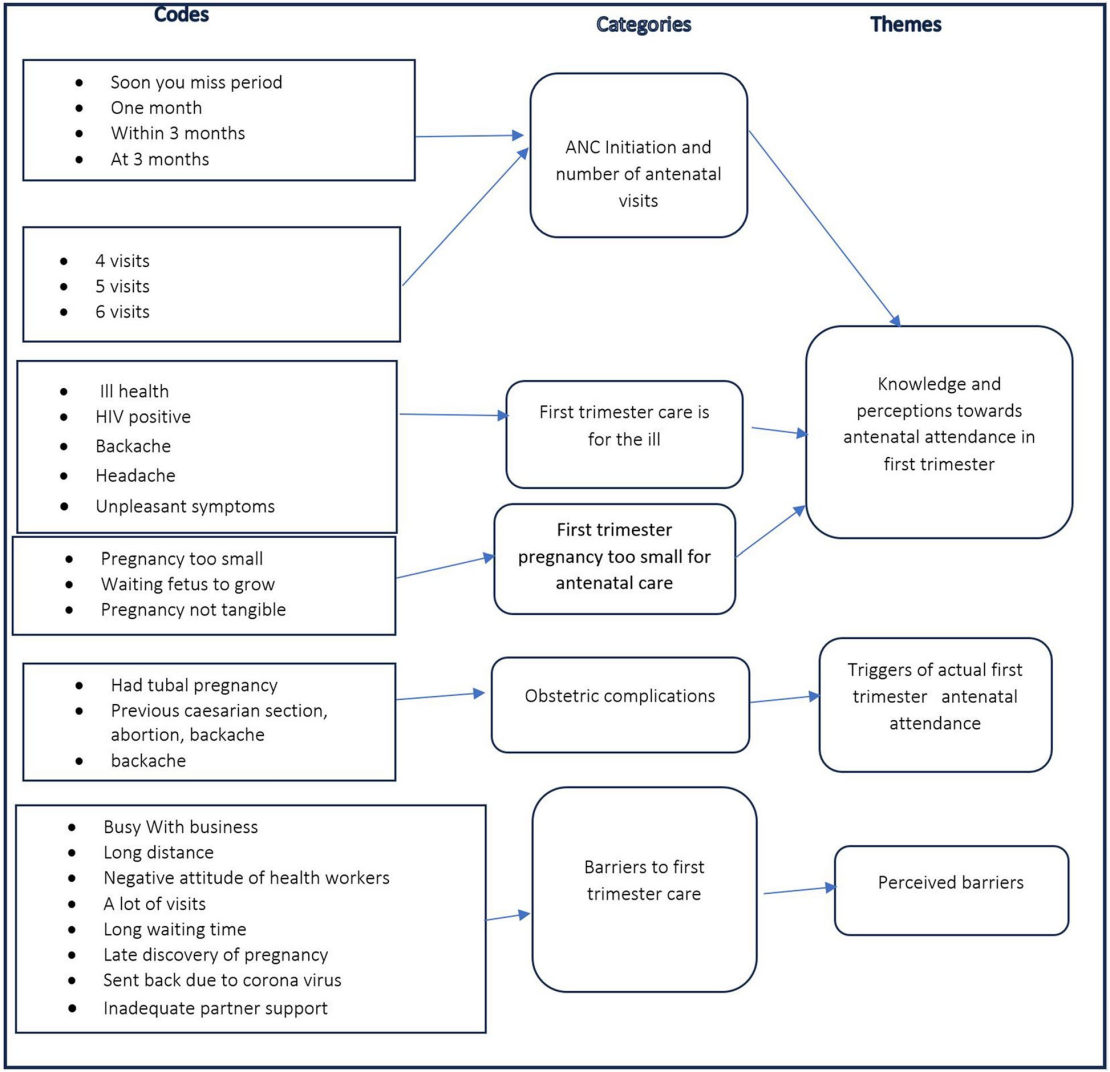
Coding and theme development

In the first phase of the thematic analysis, the researchers familiarized themselves with the data by listening to all audio recordings repeatedly. This was followed by transcription verbatim in Chichewa, which was later translated to English. Transcribed data were verified by re-reading while listening to the recorded data by MN and LN. The team used the *Chichewa* and English versions simultaneously to avoid misinterpretations of the full meaning of the texts and corrected areas that were incomplete or incorrectly transcribed. The second phase involved generation of the initial codes. The initial codes originated from the questions from the interview guide, study objectives and the HBM. We did open coding deductively. There was a predefined list of coding categories such as initiation time, dangers, benefits, and barriers and we added other codes along the way. We read transcripts repeatedly to identify patterns in the data and collated the codes with extracts. In the third phase of thematic analysis, the researchers verified the codes and extracts before categorizing. The researchers then assessed groups of codes further to identify potential themes. The researchers reflected and compared the findings between the different IDIs and FGDs to reach agreement on the codes, categories and themes. They developed a coding tree that had all codes, categories, and identified themes (Fig. 2) and used deductive theme identification approach based on the study objectives and the HBM.

In the fourth phase, the researchers reviewed potential themes in relation to coded extracts and the entire data set. There was also a validation of individual themes in relation to the data set to make sure that themes accurately reflected the meanings of the data set. In the fifth phase, they defined each theme clearly by identifying the essence of the theme and determining what aspect of data and research questions fell under the theme. Finally, the authors developed three themes from the data, namely; (a) knowledge and perceptions towards antenatal care attendance during the first trimester; (b) triggers of actual first trimester antenatal attendance and (c) perceived barriers to antenatal care attendance during the first trimester. The themes responded to the study objective, which aimed at exploring the pregnant women’s perceptions and how they influence antenatal care visit during the first trimester.

The study set out to achieve the following objectives: (a) to explore the extent to which pregnant women perceive antenatal care visits during their first trimester of the pregnancy; (b) to explore pregnant women’s perceived barriers to antenatal care visits during the first- trimester and finally, (c) to explore the link between pregnant women’s perceptions towards antenatal care visits during the first trimester and their actual attendance during the first trimester. Thus, the above objectives informed the deductive data analysis approach and the process of deducing the themes from the study. The objectives also informed the discussion of the study findings as the discussion is anchored around the developed themes, which are initially informed by the study objectives.

### Trustworthiness of the study

The following were the dimensions used to ensure trustworthiness in the study, namely; credibility, confirmability, dependability, reflexivity, and transferability [14, 19, 20].

*Credibility (internal validity)* is concerned with how congruent the findings are with reality. Approaches that enhanced credibility were rapport building, member checking, triangulation of data sources, and saturation of data [14]. We established a good rapport with participants by greeting them in a friendly manner before introducing the researcher and data collectors. Interviews were conducted in a separate room and ensured an informal, non-authoritative atmosphere. The researchers explained the study objectives in simple language for easy understanding and encouraged participants to be honest during IDs and FGDs. The team used open-ended questions to gather rich data carried out member checking during and after each interview, which involved asking the participants to confirm the data obtained during data collection. The researchers further employed various data collection methods, including FGDs, IDIs, and diary notes, to check the consistency of findings and capture the nonverbal communication. The presentation of findings included literal statements and quotations from participants to improve credibility.

*Confirmability (objectivity*) is the neutrality of the researcher in interpreting findings [19]. The researchers developed an audit trail which was a recording of activities so that others can follow [21]. These included data collection and analysis procedures throughout the study to provide evidence [22]. The audit had raw data (tape-recorded data, diary notes), data reduction and analysis notes, process notes, and data reconstruction products such as a draft of the final report to allow an independent auditor to come and make conclusions about data.

The use of the audiotape recorder for IDs and FGDs, and reviewing responses together with participants, distinguished participants’ responses from views of the researchers [22]. The research team brought different perspectives to the data analysis and interpretation process, which enhanced confirmability.

*Dependability (reliability*) refers to the degree to which a study can be duplicated and if members of the research team agree on what they see and hear when more than one observer is present [19]. In this study, dependability was achieved by the audit trail [22]. An audit trail is created by (a) describing the study’s specific purpose; (b) discussing how and why participants were chosen for the study; (c) describing how the data were collected and how long the data collection took; (d) describing how the data were reduced or transformed for analysis; (e) discussing the interpretation and presentation of the research findings; and (f) communicating the specific techniques used to determine the data’s credibility [23]. The researchers addressed the dependability issue by reporting in detail all processes within the study, hence allowing other researchers to repeat the work. The processes included: research aim, methods and their application, detailed collection of data, analysis, results, and discussion [24]. The comprehensiveness of the report will allow readers to develop a thorough understanding of methods and their effectiveness.

*Reflexivity* is the recognition and investigation of how one’s beliefs and experiences may influence the research process, including participants’ responses, data collection, interpretation, analysis, and presentation [19]. During the interviews, the researchers checked the comprehension by reflecting and checking the understanding of respondents using remarks like ‘so, if I’m hearing you correctly, you’re saying’. This provided space for meanings to be clarified and developed and to gain further explanation by the participants. To ensure this was occurring, the researchers listened to the audio-recorded interviews to ensure that they were asking questions and responding appropriately, and not leading the interviews.

Dairy notes regarding feelings, biases, and insights were written to raise awareness of the influences during data collection, analysis, and interpretation [23]. For instance, MN had a feeling that pregnant women in urban areas were educated and would likely initiate ANC in the first trimester. She ensured that her training as a nurse/midwife did not blur her data collection and interpretation lenses by imposing her values. Furthermore, LN, an experienced researcher in qualitative designs and methods reviewed the whole research process and questioned where necessary. In addition, the team transcribed the interviews verbatim and no spoken detail from the audio recordings was omitted. Transcripts were checked alongside the audio recordings to enhance the auditability of the analysis. However, it should be noted that while the researchers employed reflexivity to the best of their ability, one’s values may not be completely ruled out in research. The researchers have endeavored to open up on what they did so the reader can make sound judgment on how to use the study findings.

*Transferability* (generalizability) is the applicability of findings to other contexts and is achieved through the thorough description of study context and assumptions [19]. In this study, transferability was achieved by providing a thorough description of data in the research report to allow the reader to determine the applicability of findings. The researchers provided a thick description of the research methods, analysis, and results so that the reader may judge the research evidence’s similarity to their situation.

## Ethical considerations

We obtained ethical approval from the College of Medicine Research and Ethics Committee (COMREC), an institutional review board of Kamuzu University of Health Sciences in March 2021 (reference number P.11/20/3206). The team obtained verbal and written informed consent from individual participants prior to their participation in the study and potential study participants were free to withdraw from the study at any time. Participants were identified through code numbers during data manipulation and analysis to maintain privacy and promote anonymity.

## Findings

The findings are divided into two parts, namely; the socio-demographic characteristics of study participants and the three themes, which are “knowledge and perceptions towards antenatal attendance during first trimester”, “triggers of actual first trimester antenatal attendance”, and “perceived barriers”.

### Socio-demographic characteristics of study participants

Out of 55 respondents, seven started ANC in the first trimester while the rest (48) started after first trimester. In this study, at least half of the participants [25] had reached secondary education. 17 of the participants started antenatal care later than the first trimester and were within the age range of 18–22, while eight were in the age range of 28–32. However, out of the seven participants who initiated antenatal care in the first trimester, three were in the age range of 23–27. Furthermore, five participants who started antenatal care in the first trimester were married while 2 were single. In addition, three participants started antenatal care in the first trimester among the 40 participants who participated in the FGDs, while 4 out of 15 participants in the IDIs started antenatal care in the first trimester (Table 1).

**Table 1:** Socio-demographic characteristics of study participants, *N* = 55

Characteristics	Category	Number (N)
Age in years	18–22	18
	23–27	11
	28–32	19
	33–37	7
Marital status	Single	4
	Married	51
Religion	Christians	48
	Moslems	7
Education	No education	1
	Primary	26
	Secondary	28
	Tertiary	0
Occupation	Housewife	23
	Business	25
	Subsistence farmer	4
	Employed	3
Gravidity	1	14
	2	12
	3	13
	4	11
	5	4
	6	1
Parity	0	14
	1	12
	2	12
	3	12
	4	4
	5	1
Previous mode of delivery	Operation	3
	Normal	52
	Breech	0
Gestation at the initial visit	First trimester (0–12 weeks	7
	Second trimester (13–27 weeks)	40
	Third trimester (28–36 weeks	8

### Themes

#### Knowledge and perception towards antenatal care attendance during first trimester

The participants indicated their knowledge and perceptions of antenatal care in most of their sentiments. These are presented through subthemes of ANC initiation and number of visits; first trimester antenatal care is for the ill and first trimester pregnancy “too small” for antenatal care.

#### ANC initiation and number of visits

Through IDIs and FGDs most participants stated that the timing for ANC was as soon as they noticed they are pregnant or anytime in their first trimester. During the discussion, participants mentioned that any pregnant women should start antenatal care within 3 months or any time she realizes that she is pregnant. When asked why it was important to start ANC during the first trimester, most women mentioned the following reasons: to know their maternal (gestational/pregnancy) health status and that of the developing fetus; to prevent mother to child transmission of infections like HIV; to know the presentation of the baby; and to get vaccinations against tetanus. Other reasons were: to access counselling; Iron tablets; sulfadoxine-pyrimethamine; a mosquito net to protect them against malaria and early detection and management of pregnancy related health complications.

The following statements from FGDs demonstrate this knowledge:

*P.1. “A pregnant woman should start ANC when she has discovered that she is pregnant so that she knows her health status in terms of HIV and syphilis. The infections should not be transmitted to the unborn child and also if the pregnancy has been implanted on the right place.”*

*P.4.” We (pregnant women) are supposed to start antenatal care within 3 months of pregnancy so that we can get appropriate treatment and care. For example, we get counseling about nutrition in pregnancy and also, we receive iron tablets to prevent us from anemia and vaccines against tetanus.”*

P. 8. “*Pregnant women should start ANC within three months in order to have an HIV test and prevent the unborn child from acquiring the virus.*”

*[Agreed by all participants]. (FGD1).*

Similarly, the following were the views from IDIs:

*“Pregnant woman should start ANC within three months of pregnancy to get a mosquito net which protects us from mosquito bites, vaccines against tetanus and other drugs but I have forgotten the names.” [IDI 1,30 years, G6P5].*

*“When you start antenatal care in the first trimester, as I have done, this is the third month, you are protected from diseases because of the services you get such as HIV test, vaccination against tetanus and Fansida (sulfadoxine- pyrimethamine) against malaria.” [IDI 3,33 years, G4 P2 plus 1 previous tubal pregnancy].*

*“Pregnant women should start antenatal care at 3 months to know their health status, get iron tablets, mosquito net and Fansida (sulfadoxine- pyrimethamine) against malaria. But I have started antenatal care at 7 months because I stay very far from the hospital about 8km”. [IDI 10, 21 years, G2 P1].*

Although, most women stated that the ideal time to start antenatal care was within 3 months, there were some women who stated that 4 or 5 visits were ideal for the entire pregnancy. Some participants said that five visits were adequate for the health workers to check the health status of the unborn child. They noted as follows:

*“A pregnant woman is supposed to have four antenatal visits. You are given appointment dates on alternate months.” [IDI 2, 20 years, married G2 P1].*

*“The total antenatal visits will be seven. Pregnant women are given monthly appointments especially when they start ANC at three months.” [IDI,9, 26 years G3P2].*

*“A pregnant woman is supposed to have five visits. The visits are adequate for the entire pregnancy as health workers are able to check on the health status of the unborn child.” [IDI, 14, 35 years G5P4].*

### First trimester antenatal care is for the ill

Despite most the of participants stating that pregnant women should start antenatal care within 3 months of pregnancy and benefits for doing the same, this study found that only a few (seven out of 55) actually started their antenatal care during the first trimester. When prompted to understand their action, which did not resonate with their knowledge, women in this study explained that first trimester antenatal care was for those with ill health such as those with HIV and on Antiretroviral therapy (ARVs) so that they can protect the unborn child from contracting the virus, and those with backache and headache. Some participants stated that pregnant women who are sick so often, such as those with general body pains, should start antenatal within 3 months so that they can be assisted and given treatment as early as possible.

For instance, some participants had this to say:

*P.17. “An individual who is sick so often should start ANC early at least within three months and those who are not sick can start anytime, may be, to get vaccines such as tetanus.”*

*P.19. “Pregnant women should start antenatal care in the first trimester if they are HIV positive so that they can protect the unborn child from contracting the virus. When you are sick so often, your body becomes weak. There is need to receive help early because a pregnant woman is supposed to be healthy and strong..*

P.20. “*We should start ANC at one month as pregnancies come in different ways because others are on ARVs (Antiretroviral drugs) so when we visit the ANC clinic you are taught the truth about your pregnancy and your health.”*

*[Most participants nodded] (FGD2).*

Similar views were expressed during IDIs as follows:

*“I was having headache, and backache that’s why I came early for ANC services.” [IDI 6, 23 years GIPO].*

*“If you have problems then you can start in the first trimester like general body pains, backache. If you do not have any health problem, you may start antenatal care in the first trimester if you may wish.” [IDI12, 35 years G5P4].*

### First trimester pregnancy “too small” for antenatal care

Most participants perceived that the pregnancy during first trimester was too small for antenatal care. They indicated that the doctors will not be able to palpate anything when the women seek antenatal care services within 3 months of the pregnancy. Some participants stated that they have to wait for the fetus to grow until fetal movements are felt for them to start antenatal care. Participants stated that fetal movements make them confident that they are really pregnant and that the doctors will be able to palpate something.

Some of the respondents during interviews and discussions expressed the following views:

*“The pregnancy is too small at one or two months to start ANC because doctors will not be able to examine properly.” [IDI, 05, married, 23 years, G2P1].*

*“I started ANC at six months (laughs). I was waiting for the fetus to grow. I was wrong because I did not follow the health protocols.” [IDI, 11, 27 years].*

*“I feel that within three months, the pregnancy is very small and it’s too early to start antenatal care. When you feel fetal movements it’s when you really know that you are pregnant.” [Participant, 38, FGD4].*

### Triggers of actual first trimester antenatal care attendance

In this study, six out of the seven participants that had started antenatal care in the first trimester, presented themselves with pregnancy related health problems. The participants mentioned that they started ANC during first trimester because of bad obstetric histories, which included previous tubal pregnancy, caesarian section, neonatal death, backache and headache. Pregnant women indicated that these complications triggered them to start antenatal early so that they could be assessed properly.

Some of the respondents during IDIs said that:

*“I have started ANC early, this is the third month because the previous pregnancy was in a tube (ectopic pregnancy), and I ended up having an operation. I have come early so that I should be assessed if there are any problems.” [IDI, 3, married 33 years].*

*“This is my first pregnancy, I was having a headache, and backache that’s why I came early for ANC so that I should be assisted.” [IDI, 06, married, 23 years, G1P0].*

When other women who did not start ANC at first trimester shared their perceptions, here is what some of them said:

*“I started antenatal care at six months and today I am eight months pregnant. Those who are sick frequently such as headache and backache are the ones who are supposed to start ANC in the first trimester, if they do not become sick, there is no need of starting ANC in the first trimester.” [IDI, 13, married, 26 years].*

## Perceived barriers to antenatal care attendance during the first trimester

The perceived barriers to antenatal care attendance were grouped into health systems barriers, individual barriers and economic barriers.

### Health system barriers

These included long-distance to the facility, scheduling of antenatal visits, negative attitude of health workers, long waiting time and adherence to COVID 19 preventive measures.

### Long-distance to the health facility

Travelling beyond seven to 20 kms (long distance to health facility) to access ANC services, as indicated by the participants during both the IDs and FGDs, was another perceived health system barrier. The participants explained that they did not have money to use for transport hence postponing first-trimester antenatal care. Furthermore, participants expressed that the health center is far compared to where they reside, which they cannot manage to travel by foot. The common means of transport which they used were motorcycles and minibuses. The participants further stated that such long distances required them to have extra money to buy food, such as snacks, while waiting to be served at the facility. They had the following to say:

“*We started antenatal late because some of us stay far from the hospital and transport was a problem, it’s more than seven km, we do not have money, we are poor.”[FGD3, married, 28 years, G4P3].*

“*I do have problems with transport, I stay far from the hospital, and I do not have money for transport and money to buy food such as snacks at the clinic” [IDI, 9, 26 years, G3P2].*

*“In our location distance hinder us to start first trimester attendance it’s about 20km, and I came on the motor bike escorted by my husband”. [IDI, 13, 26 years, G2 P1].*

However, it was observed that some pregnant women who resided close to the facility also started ANC after the first trimester. After probing, participants cited deliberateness, laziness, and the preference for fewer antenatal visits as some of the reasons for the delay.

For example, some respondents during the IDIs said that:

*“In our location, we do it deliberately and due to laziness; there is nothing which can prevent someone to start antenatal care early.” [IDI, 01, married, 30 years, G6P5].*

*“In our community, women are just lazy. They just say, ‘I will go later.’ We prefer to have few visits (Laughs). We think we are ok when we are not feeling anything abnormal.” [IDI, 12, 35 years, G4P3].*

### Scheduling of antenatal care clinic days

During the interviews and discussions, some women perceived scheduling of separate days for the women on initial visit, from those coming for the subsequent antenatal care visit as deterrent to antenatal care in the first trimester. The women indicated that some healthcare providers at the antenatal clinic sent them back when they came on a day such services were not provided. The participants noted that such behaviors discourage them from accessing ANC in the first trimester. The women suggested that the health workers should be allowing them to access services any day because, when they are sent back, they fail to come back due to other constraints like money for transport fare. They narrated as follows:

*“I was sent back last month, but I came from very far (X location). Midwives said new visits do not come today, go back and come tomorrow’. My thinking was any day one can initiate ANC and it was very difficult for me to come back the following day due to transport problems.” [IDI, 10, married 21 years, G2P1].*

Similarly, participants in FGD 3 had the following views:

*P. 21. “For me this is the third month for my pregnancy. I came yesterday and I was sent back. I was told to come today because yesterday was not a day for new visits”.*

*P.23. “Mmmh, at this facility, they send you back when you come a day when it’s not for new visits and it becomes difficult to find extra money for transport fare to come on the stated days. You have to wait until you find money again for transport, leading to delays for initiating antenatal care. When we are sent back, we might have unknown complications, which may put us in danger.”*

*[All participants nodded] (FGD 3).*

### Negative attitude of healthcare providers towards women

Some participants perceived the attitude of health workers as one of deterrence toward the initiation of antenatal care in the first trimester. Participants complained that they were mocked when health workers could not palpate anything on the abdomen, as if they were pretending to be pregnant. In addition, participants stated that health workers shouted at them when they delayed putting a wrapper on the bed before lying on the examination couch. Participants stated that health workers behaved as if they had ever quarreled before, especially in the way they talked to them.

The participants had the following views during FGDs:

*P.33 “When we delay to put a wrapper (Chitenje) on the examination couch before lying down and the other wrapper to cover ourselves. Health workers at the antenatal clinic shout at us as if we have ever quarreled before. They are harsh and rude. We fail to confront them because we are afraid that they may not assist us.”*

*P.35 “Health workers tell us to bring two pieces of wrappers when entering the examination room. One to put it on the examination coach, then the other wrapper to cover yourself. We are poor, we cannot afford to do that.”*

*P.38 I was mocked by the nurses at the antenatal clinic because they could not palpate anything on the abdomen. I felt it was an insult because it was like I was pretending to be pregnant. I was told to go and buy pregnancy test kits to confirm if I was really pregnant. Nurses need to be merciful.”*

*[All participants agreed, FGD 4].*

### Long waiting time at the antenatal clinic

The majority of the participants explained that they waited for a long time at the facility to be assisted due to the high number of pregnant women. They added that they reported to the facility at 6 a.m. only to go home around 2 p.m. and that the long lines demoralize them. The participants who came for the first antenatal care visit expressed more delays due to the different stages they had to go through, like HIV, syphilis tests, and the history taking. Furthermore, participants were discouraged from starting antenatal care early to avoid such situations. Participants indicated that the facility had inadequate human resources compared to the number of women at the clinic; for example, one health worker had to check blood pressure for all the pregnant women who came to the facility. They put it this way:

*“We also get tired because we report at the facility around 6 am but health workers assist us very late. They start work around 10 am, which discourages us from starting ANC early.” [IDI, 9, 26 years, G3P2].*

“At *this facility (Ku koleji kuno), there are high volumes of women attending ANC. So, even if you come in the morning, you are attended to, late; may be around 2pm and go home in the late afternoon.” [IDI, 3, 33 years, G4 P2*^*+1*^*].*

*I always find a lot of women at the clinic, as a result, I skip some of the visits; the long queues as discouraging. This is my first pregnancy.” [IDI, 6, 23 years, G1P0].*

### Adherence to COVID-19 containment measures

Some participants during IDIs and FGDs explained that pregnant women were sent back to decongest the antenatal clinic as one of the preventive measures during the COVID-19 pandemic. The participants further stated that the health workers reduced the number of women to be seen in a day to 60 as one of the COVID-19 preventive measures. Some participants indicated that they had to report at the facility as early as 5 a.m. to get a number that was below 60 after scrambling among pregnant women themselves. The pregnant women without numbers were sent back to come the other day. They therefore stated that they failed to come back the next day due to other challenges at home. They had this to say:

“*We are being sent back to come the following day as health workers have a fixed number of 60 women to see in a day due to COVID 19 restrictions. So, when we return home, we face other challenges and fail to come back.”[IDI, 4, married, 24 years, GIPO].*

*P.3. “I came early the other day, but I was sent back with other women. Nurses said they do not want congestion due to corona virus. They examined only 60 women per day, the rest were sent back.*

*P.8 “We had to come very early to pick a number below 60, we had to scramble to get the number, otherwise, you are sent back to come the other day. We were discouraged and ended up starting antenatal late.”*

[Some participants agreed, FGD1].

### Individual barriers

The individual barriers which came out from the study were late discovery of pregnancy and frequent antenatal care appointments (visits).

### Late discovery of pregnancy

During the interviews and discussions, most women indicated that sometimes they failed to start ANC in their first trimester because they did not notice early enough that they were pregnant because of irregularities in their menstrual cycles. Some participants indicated that they did not routinely experience their monthly period. As a result, it was difficult to know whether they were pregnant or not. Participants stated that they mostly noticed they were pregnant after 3 months and after experiencing pregnancy-related symptoms like fetal movements or vomiting.

Some participants during FGDs said that:

*P.24. “I did not know that I was pregnant, I was on injectable family planning method, and I was not having menses. I was just vomiting. At first, I thought it was malaria but when I went to the hospital, it’s where I was told that I was four months pregnant.”*

*P.26. “I was doubting that I was pregnant and I used not to have menses because I was on a contraceptive method. When I tested positive for pregnancy, I was in denial as it was unintended pregnancy (Inabwera mwangozi).”*

[Some participants agreed, FGD 3].

*“I have started ANC late because I was not sure whether am pregnant or not since it is my first pregnancy and just married. [IDI4, 24 years, GIPO].*

### Frequent antenatal care appointments (visits)

The study findings also indicated that most participants perceived antenatal visits as too much for them. Participants stated that they preferred fewer visits because frequent visits were not necessary for them as they would just get tired. Some participants perceived that three visits were better for them. Furthermore, some participants indicated that if they started antenatal care within 3 months of pregnancy, doctors would give them monthly antenatal appointments, which they did not want, while others were discouraged by their friends’ comments that frequent visits were boring. They explained:

*P.30. “We do not want to have many visits; better three visits mmmmh because we get tired as well as laziness, but it’s good to have five or six visits according to appointment dates given by midwives.”*

*P.33. when you start ANC in first trimester, friends discourage us, saying ‘you have started so early, so how many visits are you going to have until the road develops pot holes? (mpaka njira ikumbike?)’”. This is a local idiom stressing how frequent the antenatal visits are.*

*[All participants nodded, FGD 4].*

Similarly, during the IDIs, some participants stated that:

*“When you start antenatal care in the first trimester, you will have a lot of visits but there is no need to have such visits, we prefer to have fewer visits before delivery because we get tired. Fewer visits are better because we reduce the transport costs.” [Participant 1 IDI, 30 years].*

*“I prefer to have fewer visits, going to the antenatal clinic every month is boring and tiresome. Health workers start work late may be around 10am and we go home around 3pm. I have started antenatal clinic at six months to reduce the visits. I just attend antenatal care as a routine that once one is pregnant has to go to the clinic for antenatal care services.” [Participant 9 IDI, 26 years, G3P2].*

*“What happens is that when you start antenatal care at three months, it may mean having so many antenatal visits up to six. Doctors will tell you to visit the clinic every month. So, to avoid theses frequent visits, we start antenatal care after first trimester which is close to delivery. The visits are tiresome.” [Participant 8, IDI, 24 years, G2P1].*

### Socio-economic barriers

The socio-economic barriers were divided into: preoccupied with business and inadequate partner support.

### Preoccupied with business

Some participants perceived that it was a waste of time to wait in long lines at the clinic because they earned a living through small-scale businesses such as selling flitters *(Mandasi).* They further expressed that they were always busy with business as life in town requires them to work to find money to buy food and other necessities. Participants stated that spending the whole day without selling their products for the sake of antenatal services was like a loss, so they kept on postponing the antenatal care visit. They explained as follows:

“*In our location, the most common barrier to first trimester ANC is, we are always busy with our businesses and we feel it’s a loss and waste of time to wait on long queues at the clinic. You know life in town, you have to work to find food and other things.” [IDI, 4, 24 years, married, GIP0*].

“*You know life in town; I was busy with business thinking that everything is fine with the pregnancy.” [IDI, 12, married, 34 years, G4P3].*

#### Inadequate partner support

Some participants perceived inadequate support from their spouses. Participants indicated that they expected their partners to give them money for transport and snacks so that they could start antenatal care in the first trimester. Pregnant women noted that they expected to have new wrappers (Zitenje) from their partners so that they could wear them for initial antenatal visits. In addition, participants stated that they expected to be given money to buy snacks because they were given sulfadoxine-pyrimethamine tablets as malaria prophylaxis to take right at the clinic, which makes them feel dizzy. However, the participants reported that their partners complained of high expenditures in the family, as a result, pregnant women postponed the initiation of antenatal care until they were provided with such resources. They noted as follows:

“*Spouses say if you start ANC early, it means expenditure will be high because the number of visits will increase. We also need money to buy a snack because we are given sulfadoxine-pyrimethamine, an intermittent preventive treatment of malaria in pregnancy, to take at the clinic which makes us feel dizzy.” [IDI, 02, married, 20 years, G2P1].*

*“We like putting on new wrappers each visit, so some men are poor and cannot afford, as a result, women do not report for ANC in the first trimester. At the clinic, they need two wrappers each visit, one to put on the bed and the other to cover up yourself during the examination, so women do not want to use old ones.” [FGD, 33, married, 30 years, G3P2].*

## Discussion

This study explored perceptions of pregnant women and how the perceptions influenced attendance during the first-trimester at Area 25 Health Centre in Lilongwe, Malawi. Three themes emerged from the narratives. The first theme is “Knowledge and perception towards antenatal care attendance during first trimester”, with three subthemes, namely; (i) Antenatal care initiation and number of visits, (ii) first trimester was for those with ill health and (iii) First trimester pregnancy ‘too small’ for antenatal care. The second theme is “Triggers of actual first trimester care”, with one subtheme of: (i) Obstetric complications which included previous tubal pregnancy, caesarian section, neonatal death, backache and headache.

The third theme is “Perceived barriers to antenatal care visit during first trimester care”, which were grouped into: (i) Health systems barriers (ii) Socio- economic barriers and (iii) individual barriers.

### Knowledge and perception towards antenatal care attendance during first trimester

 The participants in this study, perceived that first-trimester attendance was only for pregnant women with ill health, such as backaches and headaches, including HIV/AIDS. The participants perceived antenatal care visits during the first trimester as curative rather than preventive measures because they felt healthy and, therefore, could do without antenatal care in the first trimester. Pregnant women who felt they were healthy did not value first-trimester attendance because the pregnancy was not a disease to them, consistent with findings of other studies [26]. These perceptions might have influenced the timing of the initiation of antenatal care among pregnant women in this study, as the majority initiated antenatal care after the first trimester. This is in agreement with another study [4], which found that pregnant women who had health conditions were more likely to initiate antenatal care in the first- trimester. This might be the case due to inadequate knowledge on the significance of early ANC [27].

This study also revealed the perception that pregnancy in first trimester is too small, not palpable by the doctor and fetal movements are not yet present, therefore, it is not due to start antenatal care. This meant that participants perceived antenatal care as abdominal examination with a palpable pregnancy and presence of fetal movements. The participants perceived that they were not at risk of dangers and complications with the small pregnancy. The findings might have influenced the decisions of the pregnant women on the timing of antenatal initiation because the pregnancies are often palpable after the first trimester. The results of the study are consistent with findings of other studies [25, 28]. According to the HBM, pregnant women who do not feel they are susceptible to dangers that come with non-adherence to first-trimester attendance were likely to start antenatal late compared to those who feel are susceptible [29, 30]. Pregnant women require an understanding of how pregnancy will affect them so that they can acknowledge that they are at risk of getting pregnancy complications [31]. Therefore, low perceived susceptibility to bad pregnancy outcomes could be a driving force for initiation of antenatal care after first trimester in this subset of the population.

We also found that most women knew the recommended time of starting ANC to be as soon as they noticed they were pregnant or in their first trimester. Despite having this recommended knowledge, only seven out of 55 acted according to their knowledge. This is an indication that not all medical information given to pregnant women may be useful to them. Similar findings have been reported by other studies that women in developing countries, particularly in Sub-Saharan Africa, tend to wait to start ANC after first trimester [13].

 In the present study, some participants perceived that four or five visits were ideal for the entire pregnancy. This showed that some participants had inadequate knowledge of the recommended total number of antenatal visits. The World Health Organization (WHO) recommended a minimum of eight contact visits^5^ . For a pregnant woman to have eight antenatal contact visits, she must start antenatal care in the first trimester. Therefore, inadequate knowledge of the number of antenatal visits might have influenced pregnant women to initiate antenatal care after the first trimester. Evidence supports the notion that the eight-contact model improves outcomes for pregnant women and babies because of early identification and management of risk factors [4, 26].

### Triggers of actual first trimester antenatal attendance

In this study, most of the women [6] who initiated antenatal in the first trimester had issues, disorders and complications. Majority of the participants who initiated antenatal care during first trimester indicated that backache, previous tubal pregnancy and previous neonatal deaths triggered them to start antenatal care in the first trimester. The findings indicate that previous and current health and obstetric complications might have influenced the participants to initiate antenatal care in the first trimester. The trend of first trimester antenatal care that emerged in this study was similar to that of other studies in Nigeria, Ethiopia and Cameroon where several pregnant women started ANC after first trimester [28, 32, 33]. Evidence suggests that women with complications in the previous and current pregnancy were also associated with first trimester antenatal care than their counterparts [34].

### Perceived health system barriers to antenatal care visit during first trimester care

In the present study, negative attitude of health workers was also alluded as one of the health system barriers to ANC attendance. Participants reported that they were mocked when health workers could not palpate anything on the abdomen, as if they were pretending to be pregnant. The attitude of health workers may negatively influence pregnant women to initiate antenatal care after first trimester because most of the first trimester pregnancies are not palpable abdominally. Similarly, qualitative studies done in Guinea and Nigeria on factors affecting attendance noted that frequent visits and unfriendly attitudes of health workers discouraged women to initiate ANC in the first trimester [35]. This is in agreement with the Meta-Synthesis of qualitative studies on why women do not use antenatal services in low-and-middle income countries (LMIC), which indicated that the poor attitude of health workers was one of the barriers to first-trimester attendance, leading to delays in seeking health care [36, 37].

Furthermore, the participants perceived they were denied the antenatal services they deserved due to the Corona virus pandemic. Some participants stated that they were sent back by the health workers after a daily limit of 60 pregnant women. They were told to come back the next day to decongest the clinic as one of the preventive measures against the COVID-19 pandemic. Corona virus disease containment measures may have contributed to the low first-trimester antenatal attendance, and it was also a missed opportunity as some of the pregnant women who might have been in their first trimester were sent back. Literature also indicates that refusal of care after the daily patient limit has been reached contributes to low antenatal attendance in the first trimester [6, 38]. Similarly, Pitale [39], in the study on the effectiveness of protocol-based ANC during the COVID-19 pandemic, indicated that ANC services were challenging to offer but the services remained essential. However, a protocol-based approach was key to the management of ANC in Bangladesh amidst the pandemic, where pregnant women were told in advance to come to the clinic according to the stipulated schedule [39].

Long distance was one of the perceived barriers to the early initiation of ANC by the participants in this study. The participants perceived traveling seven to 20 km to a health facility as a long distance, which necessitated them to source money for transport. The participants reported common means of transport were motorcycles and minibuses. Long distance may limit the pregnant women’s ability and willingness to initiate antenatal care in the first trimester. The findings revealed that distance played a role in determining the timing of the initiation of antenatal care. Similarly, a study by Warri and George [32] in Cameroon reported that long distance deters pregnant women to seek antenatal care in the first trimester. Gatarayiha, Mesenge, and Munyanshongore, [40]^,^ in their mixed-method study on factors contributing to non-compliance with antenatal visits in Rwanda, also found that pregnant women who walked less than 30 minutes to the facility initiated antenatal care early.

It is interesting to note that some pregnant women staying close to the facility also started antenatal care after the first trimester. These mentioned laziness as the cause of the delay. This could be coupled with inadequate knowledge of the significance of antenatal care during the first trimester. In some related study, Ragolane also found that even women who stayed close to the facility initiated ANC late due to laziness [41]. In addition, Chen et al., [42] argued that individuals who perceive that they are at risk of a very harmful outcome usually consider all barriers as negligible.

It has been established in this study that majority of the participants perceived waiting time at the antenatal clinic as too long due to the high number of pregnant women and inadequate number of health care workers. Some participants indicated that they reported to the facility at 6 a.m. only to go home around 2 p.m. The long lines at the antenatal clinic demoralized pregnant women to initiate antenatal care in the first trimester. This has been associated with dissatisfaction and may lead to delay in initiation of antenatal care [43].

Furthermore, participants perceived the scheduling of separate days for the women on the initial visit from those coming for the subsequent antenatal care visit as a deterrent to antenatal care in the first trimester. The participants indicated that they were sent back when they came on the wrong days, and as a result, they became discouraged to come the other day for the antenatal care. The organization of the health system may influence the timing of antenatal care visits during the first trimester. However, Gong et al. [44] in southern Mozambique urged that with better-scheduled appointment visits, pregnant women no longer had to anticipate sacrificing an entire day at the clinic.

### Perceived socio-economic barriers to antenatal care visit during first trimester care

Some participants perceived that it was a waste of time to wait in long lines at the clinic because they earned a living through small-scale businesses such as selling flitters. Participants stated that spending the whole day without selling their products for the sake of antenatal services was a loss, so they kept on postponing the antenatal care visit. This meant that low socio-economic status and occupation of some participants as businesspersons influenced decisions on the timing of first ANC putting women at risk of complications. In addition, the problem of long waiting time indicate an area requiring improvement in the health system. This concurs with findings from a qualitative study in Guinea on factors affecting attendance and timing of ANC, where pregnant women were concerned with the opportunity cost of missing the whole day of selling their products because of ANC [32, 35].

We also found that participants in this study perceived that they were not adequately supported by their partners. Participants noted that they expected their partners to give them money for transport, snacks, and new wrappers (Zitenje) so that they could start antenatal care in the first trimester. The participants indicated that they waited until such support was provided. This indicates that partner support is paramount in antenatal care. The inadequate support could be due to financial constraints, as the participants explained that their partners complained of high expenditures. It also could be ignorance of the partners on the importance of antenatal care in the first trimester. The findings reveal that partner support play a role in determining the timing of antenatal care. This is in agreement with Suandi et al. [40, 45], who found that male partner involvement in pregnancy care appeared to have the greatest effect on healthcare utilization.

### Perceived individual barriers to antenatal care visit during first trimester care

Furthermore, some participants reported they were not aware that they were pregnant due to irregular or no periods as a result of contraceptive use. Some participants indicated that they did not experience their monthly periods, which made it difficult for them to know whether they were pregnant or not hence the delay in recognition of the pregnancy. The findings indicate that irregularities in the menstrual cycle may delay recognition of pregnancy hence affect timing in initiation of antenatal care. This concurs with another study [44], which found that pregnant women did not recognize early that they were pregnant because of previous use of contraception. However evidence indicates that early recognition of pregnancy was found to be associated with first trimester antenatal care visit [39].

Our study, found that most participants perceived antenatal visits as too much for them. Participants stated that they preferred fewer visits because frequent visits were not necessary for them as they just got tired. The findings indicate that the perceptions of the participants towards antenatal care during the first trimester did not conform to the medical recommendations. The World Health Organization (WHO) recommends a minimum of 8 antenatal contact visits for the entire pregnancy which is also being implemented at the facility [5]. Evidence indicates that pregnant women delay to initiate antenatal care to reduce appointments because the numerous visits are perceived as cumbersome [46].

### Strengths and limitations of the study

The present study findings add value to the scanty literature by showing that negative perceptions of pregnant women and other cross-cutting issues such as COVID-19 pandemics influence first trimester attendance rates. These results have been derived from a rich set of information, which was acquired through data triangulation. Data triangulation was achieved by employing diverse data collection methods including IDIs and FGDs with pregnant women. The study further used different data sources, namely; primigravidas and multigravidas, coming from the first trimester or not.

The exclusion of the age bracket, 15–17 years, might have limited some pregnant women in the study. However, some of their contributions have been captured by the participants in the age group 18–19 since they belong to the same teenage group. Additionally, since we had to protect privacy and keep information confidential for the participants, we only asked comprehensively about individual health problems during IDIs and not during FGDs. Despite the case, our structural methodology employed the five trustworthiness study dimensions; credibility, confirmability, dependability, reflexivity, and transferability to take care of information underrepresentation.

Nevertheless, this study has made significant contribution to body of literature by improving the qualitative empirical evidence regarding the perceptions of pregnant women towards antenatal care attendance during the first trimester in Lilongwe, Malawi. Moreover, it does not only address the perceptions of pregnant women, but it also identifies the triggers and barriers to antenatal care visits during the first trimester. Hence, the study findings are reliable to inform health policymakers on how to consider quality delivery of ANC services in the first trimester in Malawi.

## Conclusion(s) and recommendations

The study concludes that majority of the pregnant women perceived that the first-trimester antenatal care visits are only for those experiencing ill health conditions like backache, headache, and HIV/AIDS during pregnancy. Furthermore, first trimester pregnancy was perceived as too small and not worthy of seeking antenatal care for. The perceptions of pregnant women coupled with health systems, socio-economic and individual barriers may have influenced attendance during the first trimester. In addition, the participants had limited knowledge about the required total number of ANC visits. Addressing knowledge gaps and overcoming barriers related to economic, individual and health care delivery can improve women’s early antenatal care visits.

The study findings give room for health programs to deliver health promotion messages to the whole community, about antenatal care in the first trimester. Such community targeting would enable couples and individuals to make informed decisions before they become pregnant. In addition, health professionals and policy makers may use the findings in the refinement of antenatal care guidelines hence contributing to the reduction of maternal and neonatal mortality in Malawi. The study further recommends that health managers should plan and implement quality essential services like first-trimester antenatal care during pandemics like COVID-19. Health care managers should train healthcare workers on respectful maternity care so as to create an environment that will motivate pregnant women to initiate antenatal care in the first trimester. Future research should consider the pregnant women from diverse socioeconomic backgrounds to gain a better understanding of these perceptions and barriers.

## Data Availability

The data generated from the current study are not publicly available because we are still writing up from it, but will be available from the corresponding author on reasonable request.
